# Molecular Characterization of Dengue Type 2 Outbreak in Pacific Islands Countries and Territories, 2017–2020

**DOI:** 10.3390/v12101081

**Published:** 2020-09-25

**Authors:** Catherine Inizan, Olivia O’Connor, George Worwor, Talica Cabemaiwai, Jean-Claude Grignon, Dominique Girault, Marine Minier, Matthieu Prot, Valentine Ballan, George Junior Pakoa, Jean-Paul Grangeon, Philippe Guyant, Christelle Lepers, Daniel Faktaufon, Aalisha Sahukhan, Onofre Edwin Merilles, Ann-Claire Gourinat, Etienne Simon-Lorière, Myrielle Dupont-Rouzeyrol

**Affiliations:** 1 Institut Pasteur de Nouvelle-Calédonie, Institut Pasteur International Network, URE Dengue et Arboviroses, Noumea 98800, New Caledonia; ooconnor@pasteur.nc (O.O.); dgirault@pasteur.nc (D.G.); minier.marine91@gmail.com (M.M.); valentine.ballan@gmail.com (V.B.); mdupont@pasteur.nc (M.D.-R.); 2 Surveillance, Emergency and Research Unit, Ministry of Health, Port Vila, Vanuatu; gworwor@vanuatu.gov.vu (G.W.); jgpakoa@vanuatu.gov.vu (G.J.P.); 3 National Public Health Laboratory, Fiji Centre For Communicable Disease Control, Suva, Fiji; tcabe25@gmail.com (T.C.); dbfaktaufon@gmail.com (D.F.); aalisha@gmail.com (A.S.); 4 Laboratoire de Biologie Médicale, Hôpital de Sia, Mata’Utu 98600, Wallis et Futuna; biologiste@adswf.fr; 5 Evolutionary Genomics of RNA Viruses, Department of Virology, Institut Pasteur, 75015 Paris, France; matthieu.prot@pasteur.fr (M.P.); etienne.simon-loriere@pasteur.fr (E.S.-L.); 6 New Caledonia Health Authorities (Direction des Affaires Sanitaires et Sociales-Nouvelle-Calédonie, DASS-NC), Noumea 98800, New Caledonia; jp.grangeon@adswf.fr; 7 WHO Country Liaison Office, Port Vila, Vanuatu; guyantp@who.int; 8 Pacific Community (SPC), Noumea 98800, New Caledonia; christellel@spc.int (C.L.); jojom@spc.int (O.E.M.J.); 9 Centre Hospitalier Territorial, Microbiology Laboratory, Dumbea-sur-Mer, Dumbea 98835, New Caledonia; ann-claire.gourinat@cht.nc

**Keywords:** dengue, phylogeny, molecular evolution, Pacific

## Abstract

Dengue virus (DENV) serotype-2 was detected in the South Pacific region in 2014 for the first time in 15 years. In 2016–2020, DENV-2 re-emerged in French Polynesia, Vanuatu, Wallis and Futuna, and New Caledonia, co-circulating with and later replacing DENV-1. In this context, epidemiological and molecular evolution data are paramount to decipher the diffusion route of this DENV-2 in the South Pacific region. In the current work, the *E* gene from 23 DENV-2 serum samples collected in Vanuatu, Fiji, Wallis and Futuna, and New Caledonia was sequenced. Both maximum likelihood and Bayesian phylogenetic analyses were performed. While all DENV-2 strains sequenced belong to the Cosmopolitan genotype, phylogenetic analysis suggests at least three different DENV-2 introductions in the South Pacific between 2017 and 2020. Strains retrieved in these Pacific Islands Countries and Territories (PICTs) in 2017–2020 are phylogenetically related, with strong phylogenetic links between strains retrieved from French PICTs. These phylogenetic data substantiate epidemiological data of the DENV-2 diffusion pattern between these countries.

## 1. Introduction

The first reports of dengue virus circulation in the South Pacific region date from the end of the 19th century [1]. Since then, dengue has become a major public health concern for Pacific Islands Countries and Territories (PICTs), causing frequent outbreaks and exerting an important human and economic burden. Dengue virus (DENV) is a single-stranded, positive-sense RNA virus belonging to the genus *Flavivirus*, family *Flaviviridae*. DENVs are subdivided into four serotypes (DENV-1 to 4). Infection with one serotype is thought to provide life-long immune protection from reinfection by the same serotype. Based on the sequence of the envelope gene (*E*), each serotype may be divided into distinct genotypes, often associated with specific geographical regions [2]. In PICTs, DENV is mainly transmitted by *Aedes aegypti,* but also by *Aedes albopictus*, and by some endemic vectors such as *Aedes polynesiensis* [3].

**Figure 1:**
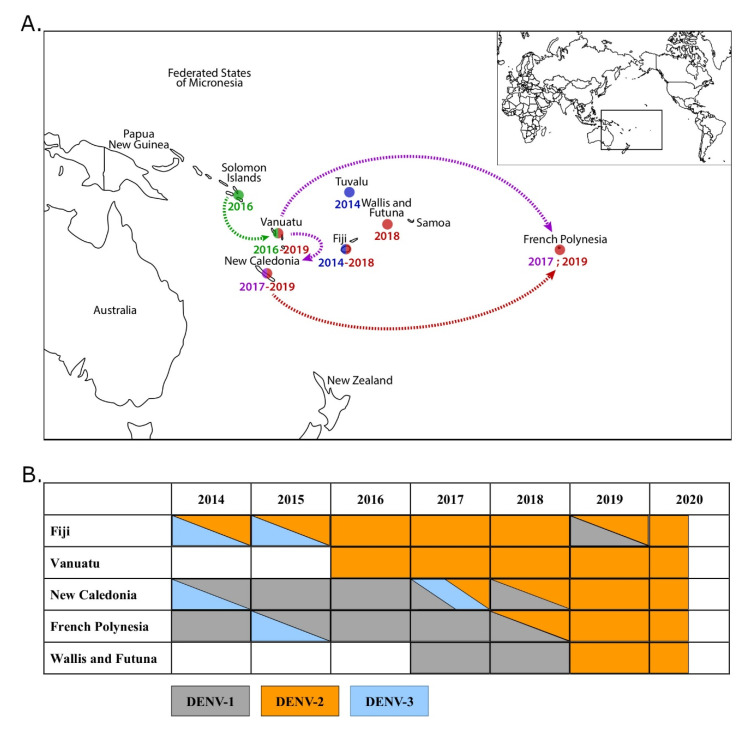
Dengue virus type 2 (DENV-2) emergence and circulation in the Pacific region, 2014–2020. (**A**) Map of the Pacific region showing the temporality of DENV-2 emergence and circulation between 2014 and 2019. DENV-2 emerged in Fiji and Tuvalu in 2014 (blue) and Solomon Islands and Vanuatu in 2016 (green). DENV-2 was then imported from Vanuatu in New Caledonia and French Polynesia in 2017 (purple), and from New Caledonia in French Polynesia in 2019 (red). Arrows indicate putative routes of introductions. (**B**) Temporality of DENV circulation in Fiji, Vanuatu, New Caledonia, French Polynesia, and Wallis and Futuna between 2014 and 2020 (according to the Pacific Public Health Surveillance Network [10]).

Since World War II, dengue circulation in PICTs used to be characterized by the sustained transmission of only one dengue serotype at a time [1]. Over the last decade, however, dengue outbreaks are becoming more frequent and involve the co-circulation of several DENV serotypes [4,5]. Before 2017, DENV-1 was the major circulating serotype in French Polynesia (FP), Wallis and Futuna (WF), and New Caledonia (NC) [4,5,6] (Figure 1B). 

The last DENV-2 outbreaks in the region occurred in 1971–1974 [1,7] and 1997–1999 [1,8]. No DENV-2 circulation was then detected in the South Pacific over 15 years. In 2014, DENV-2 re-emerged in Fiji and Tuvalu. DENV-2 circulation was further detected in Fiji in 2015–2016 [9]. In 2016, DENV-2 circulation was detected in Vanuatu and Solomon Islands [10,11] (Figure 1A). A church conference held in Port Vila in November 2016 may have led to the import of DENV-2 from Solomon Islands to Vanuatu. The outbreak in Vanuatu lasted until May 2017 with 2950 suspected cases, of which 671 were confirmed using dengue rapid diagnostic tests. In 2017, DENV-2 was imported from Vanuatu into FP [12] and NC (Figure 1A). While DENV-2 introduction in FP did not lead to an outbreak [13], its introduction in NC was followed by a co-circulation with DENV-1 and DENV-3 in 2017, with 81.1%, 17.4%, and 1.5% of the cases being attributable to DENV-1, 2, and 3, respectively. In 2018, 13.2% and 86.5% of the cases were caused by DENV-1 and DENV-2, respectively [4,14] (Figure 1B). In 2018, DENV-2 indeed became the major circulating serotype in NC, responsible for 86.5% of the 2087 cases detected [14]. At the same time, cases of DENV-2 were imported in WF from NC and Vanuatu in 2017 and 2018 (Figure 1A,B). In 2019 and 2020, DENV-2 was the only circulating serotype in NC and WF. DENV-2 has caused 3916 cases in 2019 and 52 cases as of August 2020 in NC [14]. DENV has caused 30 cases in WF in 2019 and 54 as of August 2020 [15]. DENV-2 was also detected in FP in travelers returning from NC in 2019; these introductions led to a DENV-2 outbreak of 2947 cases in FP in 2019–2020 [13] (Figure 1A,B).

Epidemiologic and molecular epidemiologic studies are paramount to decipher the epidemic patterns of DENV spread. Based on phylogenetic analyses, the current work attempts to substantiate the introduction and circulation routes of DENV-2 in PICTs between 2017 and 2020, unveiled by epidemiological studies.

## 2. Materials and Methods

### 2.1. Samples

Upon request from some Pacific Islands Countries and Territories, among which are Vanuatu and Fiji, the Institut Pasteur in New Caledonia (IPNC) performs regional molecular surveillance of DENV circulation in the South Pacific region, based on DENV confirmation and serotyping by RT-qPCR. In this context, IPNC receives twice a year a subset of sera or serum samples blotted on pre-cut circles of filter-paper cards positive for dengue nonstructural protein 1 (NS1) by rapid detection test (SD BIOLINE Dengue Duo, Standard Diagnostics, Yongin, South Korea). For the current study, these samples were collected in Vanuatu in 2018–2020 and in Fiji in 2018–2019. Furthermore, in the framework of an ongoing research collaboration with the Territorial Hospital in New Caledonia, the Institut Pasteur in New Caledonia performs DENV genotyping on a subset of serum samples positive for DENV by RT-qPCR. The Institut Pasteur in New Caledonia thus received serum samples collected in WF in 2019 and in NC in 2017–2020. Viral RNA was extracted from these samples using the QIAamp Viral RNA extraction kit (Qiagen, Hilden, Germany). Samples identified as DENV-2+ by serotyping PCR [16] were further processed for sequencing. Ethic approval for human samples analysis was granted by a Persons Protection Committee (CPP Sud-Est II n°Eudract 2019-A03114-53) and by the Consultative Ethics Committee of New Caledonia.

### 2.2. Sequencing of DENV Strains

The *E* genes from DENV-2+ samples were amplified and sequenced as described previously [16]. Overlapping fragments were assembled with Staden Package (Medical Research Council, Laboratory of Molecular Biology, Cambridge, England) [17]. Sequences have been deposited into GenBank under accession numbers MN548842–MN548855, MN566109–MN566112, and MT799878–MT799882. 

### 2.3. Maximum Likelihood Phylogenetic Analysis

Nucleotide sequences were aligned with Clustal W integrated in Bioedit version 7.2.5 software [18]. A maximum likelihood phylogenetic analysis was carried out with MEGA version 10.0 software [19], using 89 *E* gene nucleotide sequences, among which 66 were retrieved from GenBank and 23 were generated in the current study. Sequences retrieved from GenBank encompass all 5 DENV-2 genotypes. Sequences from the last DENV-2 outbreak in the South Pacific were retrieved (1997–1999), along with recent (2010–2019) sequences from the Pacific and South East Asia. The evolutionary distances were computed using the Tamura–Nei 93-parameter method defined as the best evolutionary model with MEGA for our set of data. A discrete gamma distribution was used to model evolutionary rate differences among sites (3 categories). Partial deletion was used to treat gaps and missing data. The robustness of the nodes was evaluated by bootstrap with 1000 replicates.

### 2.4. Maximum Clade Credibility Phylogenetic Analysis

A maximum clade credibility (MCC) tree was built for 48 complete *E* gene nucleotide sequences (1448 nt) of isolates collected in the Pacific and South East Asia between 2010 and 2020, among which 28 were retrieved from GenBank and 20 were generated in the current study. A codon-partitioned model was applied using the Tamura–Nei 93 substitution model with a gamma distribution. Different model combinations were tested, using either a strict clock, an uncorrelated relaxed clock with a lognormal relaxed distribution, or a relaxed clock with an exponential relaxed distribution. A constant, or a Bayesian Skygrid estimate of the population size, was applied. Path sampling (PS) and stepping stone sampling (SS) marginal likelihood estimates (MLE) were computed for these models (Table S1). The lowest MLE were obtained for a constant population size and a relaxed clock with a lognormal relaxed distribution. This model was further used to generate the MCC tree. Both mixing of individual chains and sufficient effective sample size were ensured by running each data for 20M generations, sampling every 10k generations using BEAST v1.10.4 [20]. After discarding 10% burn-in, a consensus file was produced using TreeAnnotator (BEAST package) and was visualized using FigTree 1.4.4.

## 3. Results

**Figure 2:**
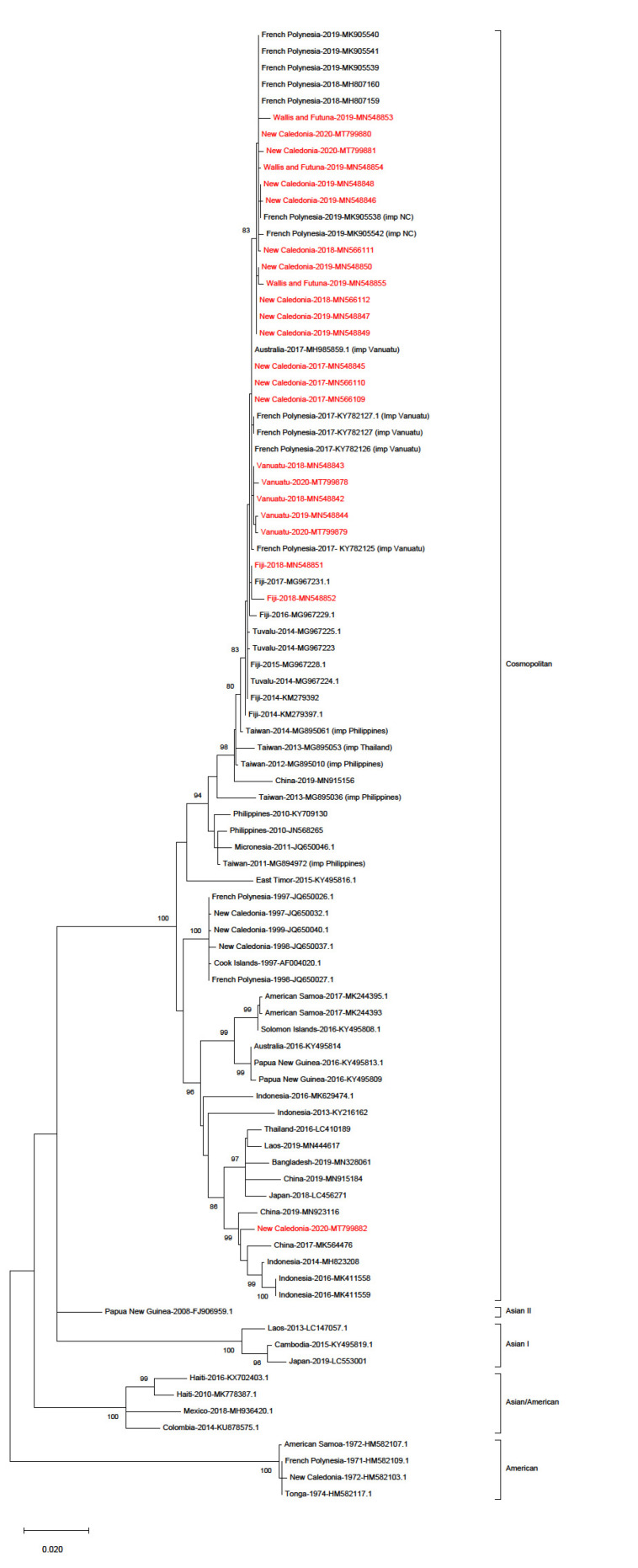
Maximum likelihood evolutionary (MLE) relationships of DENV-2 *E* gene sequences. Maximum likelihood tree derived from 89 DENV-2 *E* gene sequences (23 from Fiji, Vanuatu, Wallis and Futuna, and New Caledonia in red, and 66 from the 5 genotypes retrieved from GenBank, including isolates collected in 2010–2019 in the Pacific and in South East Asia). The percentage of replicate trees in which the associated taxa clustered together in the bootstrap test (1000 replicates) is shown for values over 80. The country and year of collection are indicated along with the GenBank accession number; imp = imported from.

DENV-2 strains collected in Fiji, Vanuatu, WF, and NC between 2017 and 2020 belonged to the Cosmopolitan genotype, which was responsible for the last DENV-2 outbreak in the region in 1997–1999. While 1448 nt of the *E* gene could be sequenced from 21 samples collected in NC, WF, and Vanuatu, only 716 nt could be amplified and sequenced from the two samples collected in Fiji in 2018. Phylogenetic analyses based on *E* gene sequences obtained from these 23 samples indicated that these strains, along with strains retrieved in FP, formed a single cluster, except for one strain retrieved in New Caledonia in 2020. This cluster shared a common ancestor with strains detected in Fiji and Tuvalu in 2014 (Figure 2).

**Figure 3:**
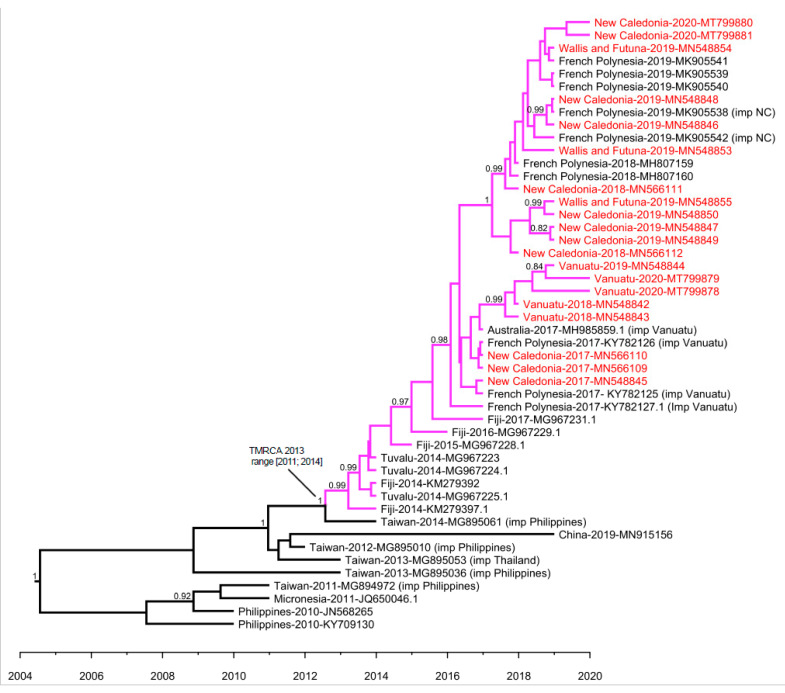
Maximum clade credibility (MCC) evolutionary relationships of DENV-2 *E* gene sequences from the Pacific and South East Asia. Maximum clade credibility tree of 48 complete *E* gene nucleotide sequences of isolates collected in Pacific and South East Asia, among which 20 were generated in the current study. The two shorter sequences retrieved in Fiji, as well as the sequence “New Caledonia-2020-MT799882”, which does not cluster with the other strains retrieved in the South Pacific region in 2017–2020, were excluded from the analysis. This MCC tree was obtained for a constant population size and a relaxed clock with a lognormal relaxed distribution. This model yielded the lowest MLE. For clarity, the tree displays all South Pacific virus branches in purple. The 20 virus sequences obtained in this study are indicated in red. Posterior probabilities higher than 0.80 are specified for each node; TMRCA = Time to the Most Recent Common Ancestor.

Strains retrieved in the South Pacific region in 2014–2020 shared a common ancestor with a strain retrieved in China in 2019, a strain retrieved in Micronesia in 2011, and strains originating from the Philippines and Thailand, collected between 2010 and 2014. Our Bayesian analysis estimated that the most recent common ancestor of strains circulating in the South Pacific region (Figure 3, purple subtree) dates from 2013 (range 2011–2014). The sequence of the ancestor of all strains retrieved in the South Pacific between 2014 and 2020 was determined by ancestral state reconstruction (Figure S1). The best hits upon blasting of this ancestral sequence were found to be a DENV-2 sequence retrieved in Fiji in 2014 (GenBank KM279394.1) with 100% identity, another sequence retrieved in Fiji in 2014 (GenBank KM279392.1) with 99% identity, and a sequence imported from the Philippines in China (GenBank MG840595.1) with 99% identity.

Within the South Pacific cluster, strains collected in 2018–2020 in French PICTs, namely NC, WF, and PF formed a sub-cluster, supported by a bootstrap value of 83 and a posterior probability of 1. Amino acid sequences of these strains were highly homogeneous, with variation between them detected only at four positions (12, 21, 46, and 200). This sub-cluster was distinct from strains collected in NC in 2017, or collected in and imported from Vanuatu in 2017–2020. This sub-cluster contained DENV-2 strains imported from NC in FP in 2019. Both maximum likelihood and MCC analyses suggested that strains collected between 2018 and 2020 in Vanuatu formed a cluster within the South Pacific cluster, distinct from strains circulating in French PICTs.

Fiji DENV-2 strains from 2018 were very close to a DENV-2 strain collected in Fiji in 2017. Amino acid sequences of DENV-2 strains collected in Fiji in 2014–2018 were identical except for the sequence KM279397 collected in Fiji in 2014, which showed a valine at position 347, whereas all other Fiji strains showed an alanine.

Surprisingly, a DENV-2 strain retrieved in NC in 2020 clustered with strains collected in China in 2017 and 2019, and in Indonesia in 2014 and 2016 (Figure 2). This case had no reported history of recent travel. Finally, DENV-2 strains retrieved in Solomon Island in 2016 and American Samoa in 2017 also belonged to the Cosmopolitan genotype, albeit to a different lineage (Figure 2).

## 4. Discussion

Following a 15-year period during which no DENV-2 circulation was detected, DENV-2 re-emerged in the South Pacific in 2014, when herd immunity for DENV-2 was low [21]. DENV-2 circulation in the South Pacific region between 2014 and 2020 was imputable to the Cosmopolitan genotype, and the most recent common ancestor to all South Pacific strains was estimated to date from 2013. The representativity of publicly available genomic data likely prevented the reliable inference of the geographic origin of this DENV-2. South Pacific DENV-2 strains, however, were phylogenetically close to sequences from South East Asia (SEA), which suggested a possible introduction from this region. Multiple population exchange between PICTs and SEA, both for tourism and trade, may have favored this introduction.

DENV-2 was first detected in Fiji and Tuvalu in 2014. Ancestral state reconstruction indicated that the first DENV-2 circulating in the region was nearly identical to strains retrieved in Fiji in 2014. Both molecular and epidemiological data indicated low-noise circulation of DENV-2 in Fiji between 2014 and 2018 [9]. DENV-2 may thus have continuously circulated in Fiji between 2014 and 2018 in the absence of re-introduction.

Epidemiological data revealed that DENV-2 was detected in Vanuatu in 2016 following its likely introduction from the Solomon Islands. DENV-2 circulated in Vanuatu in 2017–2020. The cluster of Vanuatu strains collected between 2018 and 2020 suggested a local differentiation of the strain initially detected in Vanuatu in the absence of reintroduction, and which was not detected in the rest of the Pacific.

Surprisingly, despite the reported import of DENV-2 in Vanuatu from Solomon Islands, strains retrieved in Solomon Islands in 2016 did not cluster with our sequences from Vanuatu and French PICTs. This discrepancy with epidemiological data was probably due to a lack of representativity of the sampling. It nonetheless suggested the co-circulation of a different lineage of DENV-2 from the Cosmopolitan genotype in the region.

At the beginning of 2017, the first DENV-2 cases detected in NC and FP [21] were imported from Vanuatu, which corroborated the phylogenetic link observed between strains from Vanuatu, NC, and FP. The cluster of DENV-2 strains collected in 2018–2020 in WF, NC, and FP most probably reflected the frequent population exchanges between these three French overseas territories.

Although deserving further investigation to evaluate the breadth of its circulation, the detection in 2020 in NC of a distinct DENV-2 strain, clustering with strains from SEA in a patient with no recent travel history, suggested a possible local circulation in NC. Thorough molecular surveillance in the next months will allow us to determine the epidemiological importance of this lineage in NC.

Overall, our phylogenetic data substantiated the epidemiological data gathered on DENV-2 circulation in the South Pacific region between 2017 and 2020, suggesting at least three different DENV-2 introductions; these introductions were associated with the diffusion of the same strain sub-cluster in the three French PICTs within the region.

Tourist exchanges are frequent within the Pacific region, and arboviruses introduction in NC from SEA [6,22] or other Pacific islands, notably from FP or Vanuatu, has already been reported [23,24]. Pacific islands are thus exposed to viral introductions from neighboring countries, with a potential for onward spread within the region.

The epidemiological and virological determinants of DENV-2 emergence in the South Pacific region may rely on an enhanced fitness of DENV-2 over the previously circulating serotypes, or on differential cross-reactive herd immunity for the different serotypes. The current implementation of the *Wolbachia* strategy in Vanuatu, Fiji, and NC in the framework of the World Mosquito Program may reshape the dynamics of DENV serotype replacements.

## Supplementary Materials

The following are available online at https://www.mdpi.com/1999-4915/12/10/1081/s1, Figure S1: Maximum clade credibility evolutionary relationships of DENV-2 *E* gene sequences from the Pacific and South East Asia. Table S1: Path sampling and stepping stone sampling marginal likelihood estimates of tested Bayesian models.
