# Supplementary material for: Molecular Characterization of Dengue Type 2 Outbreak in Pacific Islands Countries and Territories, 2017–2020
Source: Viruses. 2020 Sep 25;12(10):1081. doi: 10.3390/v12101081 (PMC7601490; doi:10.3390/v12101081)
Supplement: Supplementary file 1 [file viruses-12-01081-s001.pdf]

viruses

MDPI

Supplementary

Molecular Characterization of Dengue Type 2 Outbreak in Pacific Islands Countries and Territories, 2017–2020

Catherine Inizan [1,*,†], Olivia O'Connor [1,†], George Worwor [2], Talica Cabemaiwai [3], Jean-Claude Grignon [4], Dominique Girault [1], Marine Minier [1], Matthieu Prot [5], Valentine Ballan [1], George Junior Pakoa [2], Jean-Paul Grangeon [6], Philippe Guyant [7], Christelle Lepers [8], Daniel Faktaufon [3], Aalisha Sahukhan [3], Onofre Edwin Merilles, Jr. [8], Ann-Claire Gourinat [9], Etienne Simon-Lorière [5] and Myrielle Dupont-Rouzeyrol [1]

1 Institut Pasteur de Nouvelle-Calédonie, Institut Pasteur International Network, URE Dengue et Arboviroses, Noumea 98800, New Caledonia; ooconnor@pasteur.nc (O.O.); dgirault@pasteur.nc (D.G.); minier.marine91@gmail.com (M.M.); valentine.ballan@gmail.com (V.B.); mdupont@pasteur.nc (M.D.-R.)
2 Surveillance, Emergency and Research Unit, Ministry of Health, Port Vila, Vanuatu; gworwor@vanuatu.gov.vu (G.W.); jgpakoa@vanuatu.gov.vu (G.J.P.)
3 National Public Health Laboratory, Fiji Centre For Communicable Disease Control, Suva, Fiji; tcabe25@gmail.com (T.C.); dbfaktaufon@gmail.com (D.F.); aalisha@gmail.com (A.S.)
4 Laboratoire de Biologie Médicale, Hôpital de Sia, Mata'Utu 98600, Wallis et Futuna; biologiste@adswf.fr
5 Institut Pasteur, Evolutionary Genomics of RNA Viruses, 75015 Paris, France; matthieu.prot@pasteur.fr (M.P.); etienne.simon-loriere@pasteur.fr (E.S.-L.)
6 New Caledonia Health Authorities (Direction des Affaires Sanitaires et Sociales-Nouvelle-Calédonie, DASS-NC), Noumea 98800, New Caledonia ; jp.grangeon@adswf.fr
7 WHO Country Liaison Office, Port Vila, Vanuatu; guyantp@who.int
8 Pacific Community (SPC), Noumea 98800, New Caledonia; christellel@spc.int (C.L.); jojom@spc.int (O.E.M.J.)
9 Centre Hospitalier Territorial, Microbiology Laboratory, Dumbea-sur-Mer, Dumbea 98835, New Caledonia; ann-claire.gourinat@cht.nc
* Correspondence: cinizan@pasteur.nc; Tel.: +687-27-26-66
† These authors contributed equally to this work.

Abstract: Dengue virus (DENV) serotype-2 was detected in the South Pacific region in 2014 for the first time in 15 years. In 2016–2020, DENV-2 re-emerged in French Polynesia, Vanuatu, Wallis and Futuna, and New Caledonia, co-circulating with and later replacing DENV-1. In this context, epidemiological and molecular evolution data are paramount to decipher the diffusion route of this DENV-2 in the South Pacific region. In the current work, the E gene from 23 DENV-2 serum samples collected in Vanuatu, Fiji, Wallis and Futuna, and New Caledonia was sequenced. Both maximum likelihood and Bayesian phylogenetic analyses were performed. While all DENV-2 strains sequenced belong to the Cosmopolitan genotype, phylogenetic analysis suggests at least three different DENV-2 introductions in the South Pacific between 2017 and 2020. Strains retrieved in these Pacific Islands Countries and Territories (PICTs) in 2017–2020 are phylogenetically related, with strong phylogenetic links between strains retrieved from French PICTs. These phylogenetic data substantiate epidemiological data of the DENV-2 diffusion pattern between these countries.

Keywords: dengue; phylogeny; molecular evolution; Pacific

Table S1. Path Sampling and Stepping Stone Sampling Marginal Likelihood Estimates of tested Bayesian models.

Population Size	Strict clock	Relaxed clock - lognormal relaxed distribution	Relaxed clock - exponential relaxed distribution
Constant	PS[1] -2869.47<br>SS[2] -2869.47	PS -2879.79<br>SS -2880.47	PS -2869.06<br>SS -2869.32
Bayesian Skygrid	PS -2865.73<br>SS -2865.90	PS -2871.37<br>SS -2871.65	PS -2864.95<br>SS -2865.18

[1] PS = Path Sampling, [2.] SS = Stepping Stone Sampling

>Ancestral state DENV-2 Pacific

ATGCGTTGTATAGGAATATCAAATAGAGACTTTGTGGAAGGGGTTTCAGGAGGAAGCTGG

GTTGACATAGTCTTAGAACATGGAAGCTGTGTGACGACGATGGCGAAAAATAAACCAACA

TTGGACTTTGAACTGATAAAAACGGAAGCCAAACATCCCGCCACTTTAAGGAAGTATTGT

ATAGAGGCAAAGCTGACCAACACAACTACAGCATCTCGTTGCCCAACACAAGGAGAACCC

AGCCTAAATGAAGAACAGGACAAAAGGTTTGTCTGCAAACACTCCATGGTAGACAGAGGA

TGGGGAAATGGATGCGGATTGTTTGGAAAGGGAGGCATCGTGACCTGTGCAATGTTCACA

TGCAAAAAGAACATGGAAGGAAAAGTCGTGCAACCAGAAAACTTGGAGTATACCATTGTG

ATAACACCTCACTCAGGGGAAGAGAATGCAGTCGGAAATGACACAGGAAAACACGGCACG

GAAATTAAAGTAACGCCACAGAGTTCCATCACAGAAGCGGAACTGACAGGCTATGGCACT

GTCACGATGGAATGCTCTCCGAGAACGGGCCTCGACTTTAATGAGATGGTGTTGCTGCAA

ATGGAAGACAAGGCTTGGCTGGTGCACAGGCAATGGTTCTTAGACCTGCCGTTACCATGG

CTGCCCGGAGCAGACAAACAAGGATCAAATTGGATACAGAAGGAGACATTGGTCACTTTC

AAAAATCCCCATGCGAAGAAACAGGATGTTGTTGTTTTAGGATCCCAAGAAGGGGCCATG

CATACAGCACTCACAGGGGCCACGGAAATCCAGATGTCATCAGGAAACTTACTGTTCACA

GGACATCTCAAGTGCAGGCTGAGAATGGACAAACTACAGCTCAAAGGAATGTCATATTCT

ATGTGTACAGGAAAGTTTAAAGTTGTGAAGGAAATAGCAGAAACACAACATGGAACAATA

GTTATCAGAGTACAATATGAAGGGGACGGTTCTCCGTGCAAGATCCCTTTTGAAATAATG

GATTTGGAAAAAAGACATGTCTTAGGCCGCTTGATCACAGTCAACCCAATTGTTACAGAA

AAAGACAGCCCAGTCAACATAGAAGCAGAACCTCCATTTGGAGACAGTTACATCGTTATA

GGAGTAGAACCGGGACAACTGAAGCTCAGCTGGTTCAAGAAAGGGAGTTCTATTGGCCAA

ATGTTTGAGACAACAATGAGAGGAGCGAAGAGAATGGCCATTTTAGGTGACACAGCTTGG

GATTTTGGATCCCTGGGAGGAGTGTTCACATCTATAGGAAAGGCCCTCCACCAAGTTTTT

GGAGCAATCTATGGGGCTGCCTTTAGCGGGGTTTCATGGACTATGAAAATCCTTATAGGA

GTCGTCATCACATGGATAGGAATGAATTCACGCAGCACCTCACTGTCTGTGTCACTAGTA

TTAGTGGG

Figure S1. Ancestral state reconstruction of the sequence of The Most Recent Common Ancestor to all strains retrieved in the South Pacific between 2014 and 2020.
